# Human chromatin remodelers regulating HIV-1 transcription: a target for small molecule inhibitors

**DOI:** 10.1186/s13072-025-00582-w

**Published:** 2025-04-16

**Authors:** Yuan Ma, Chuan Li, Susana Valente

**Affiliations:** Department of Immunology and Microbiology, The Herbert Wertheim UF Scripps Institute for Biomedical Innovation & Technology, Jupiter, FL 33458 USA

**Keywords:** HIV-1 transcription, HIV-1 latency, Epigenetics, Chromatin remodeling complexes, Tat-TAR

## Abstract

HIV-1 can establish a lifelong infection by incorporating its proviral DNA into the host genome. Once integrated, the virus can either remain dormant or start active transcription, a process governed by the HIV Tat protein, host transcription factors and the chromatin landscape at the integration site. Histone-modifying enzymes and chromatin-remodeling enzymes play crucial roles in regulating this chromatin environment. Chromatin remodelers, a group of ATP-dependent proteins, collaborate with host proteins and histone-modifying enzymes to restructure nucleosomes, facilitating DNA repair, replication, and transcription. Recent studies have highlighted the importance of chromatin remodelers in HIV-1 latency, spurring research focused on developing small molecule modulators that can either reactivate the virus for eradication approaches or induce long-term latency to prevent future reactivation. Research efforts have primarily centered on the SWI/SNF family, though much remains to be uncovered regarding other chromatin remodeling families. This review delves into the general functions and roles of each chromatin remodeling family in the context of HIV and discusses recent advances in small molecule development targeting chromatin remodelers and the HIV Tat protein, aiming to improve therapeutic approaches against HIV.

## Introduction

HIV remains a significant global health challenge, with approximately 39 million people currently living with HIV (PLWH) and an alarming 1.3 million new infections reported in 2023 [1]. Around 76% of PLWH receive antiretroviral therapy (ART), which has had remarkable success at reducing mortality and morbidity. However, despite its effectiveness, ART is not a cure, and PLWH must maintain lifelong treatment [2–5]. This necessity arises because HIV persists in a latent state within CD4^+^T cells, even in individuals who have been on ART for extended periods. These stable cell reservoirs, harboring proviral DNA, are the source of viremia when ART is discontinued [6–8].

For decades, tremendous efforts have been placed into understanding the complexities of HIV transcription within these reservoir cells, yet there is still much to learn. HIV transcription is controlled by a dynamic interplay of inducible host transcription factors (TFs), chromatin regulatory complexes (CRCs) and viral activating factors such as the HIV Tat protein. Together, these elements orchestrate the spatiotemporal control of HIV expression throughout the course of infection and in response to exogenous triggers, such as T cell activation [9]. Typically, HIV integrates into active chromatin regions, which facilitates efficient transcription of the viral genome. However, the location of integration also influences HIV expression [10], for instance, integration near certain active genes or enhancers can lead to transcriptional read through or the recruitment of transcriptional co-activators to the viral promoter [11]. In addition, transcriptional interference from neighbouring genes can either suppress or enhance HIV transcription depending on the direction and extend of their transcription [12, 13].

**Fig. 1:**
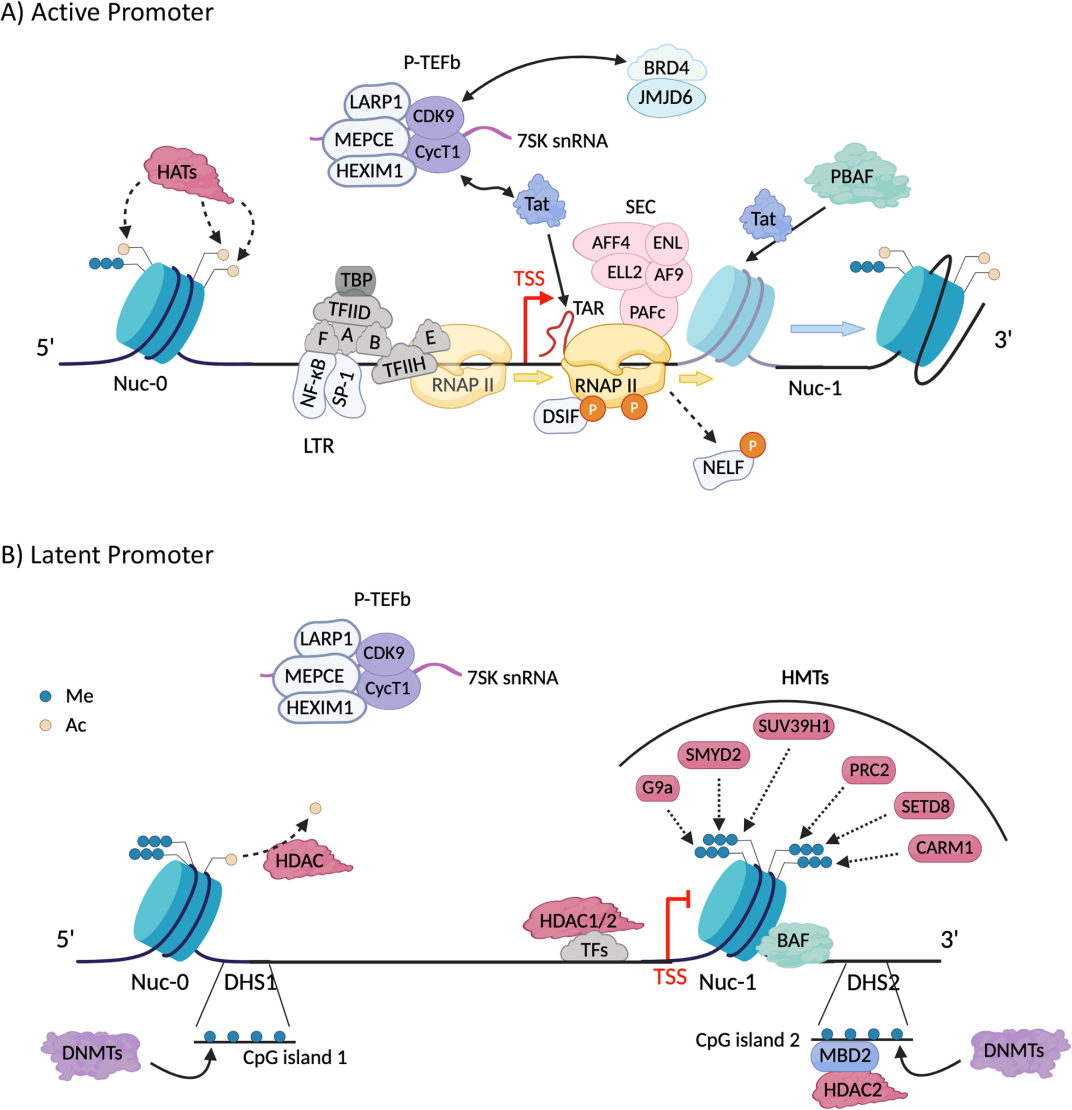
Simplified Version of Factors Recruited to the Transcriptionally Active and Latent HIV Promoter. Both 1A and 1B are adapted from reviews by Gibaut and Mori published in *Current Opinion in HIV and AIDS* in 2023 and *Viruses* in 2020 respectively. **A** Active Promoter: Upon activation, transcriptional repressors are replaced by transcriptional activators, marking the beginning of the transcription process. The initial step involves the assembly of the PIC at the promoter region. This assembly is orchestrated by the synchronized binding of general transcription factors (GTFs), including the TATA-binding protein (TBP) to the TATA box within TFIID, TFIIA, TFIIB, and TFIIF, along with the mediator complex. Additional activator TFs, such as NF-κB and Sp1, further contribute to the assembly by binding to the long terminal repeat (LTR). While PIC formation initially proceeds independently of Tat, the presence of Tat significantly enhances this process. Tat recruits TBP to the HIV-1 LTR, bypassing the requirement for other TFIID components like TAFs, through its interaction with P-TEFb. The final stages of PIC assembly involve the recruitment of RNAPII and the remaining GTFs, TFIIE and TFIIH. Under basal condition, BRD4, which belongs to the bromodomain-containing protein family (BRD), competes with the HIV Tat protein for P-TEFb binding. This competitive interaction inhibits efficient Tat-dependent transcription elongation. When Tat protein reaches a sufficient concentration, it recruits P-TEFb (CDK9 and Cyclin T1) to the transactivation response element (TAR) RNA. CDK9 acts as an early elongation kinase, overcoming promoter proximal pausing by phosphorylating Ser2 on the RNAPII CTD, as well as two elongation factors associated with paused RNAPII: NELF and DSIF. Phosphorylation of DSIF by the CDK9 subunit of P-TEFb transforms it into a positive elongation factor that accompanies RNAPII through the gene body. Conversely, phosphorylation of NELF by P-TEFb leads to its dissociation from the transcription complex, allowing RNAPII to efficiently elongate. Tat also recruits the PBAF complex, which repositions Nuc-1 further downstream of the TSS, further enhancing transcriptional elongation. Histone acetyltransferases (HATs) are subsequently recruited to acetylate histones, relaxing the chromatin structure and rendering it more accessible for the transcriptional machinery. **B **Latent Promoter: During latency, the positive transcription elongation factor b (P-TEFb), composed of CDK9 and CycT1, is sequestered in an inactive complex with the 7SK small nuclear RNA (7SK snRNA), HEXIM, MEPCE and LARP7. Several transcription factors (TFs) such as YY1 directly bind to DNA, facilitating the recruitment of histone deacetylases (HDACs) and histone methyltransferases (HMTs). HDACs remove acetyl groups from histones, while HMTs add methyl groups to histones. Additionally, the BAF complex, a member of the SWI/SNF chromatin remodeling family, positions Nuc-1 downstream of the transcription start site (TSS), in an unfavorable position for transcriptional elongation. DNA methyltransferases (DNMTs) are also thought to hypermethylate the CpG islands near the TSS, leading to the recruitment of HDACs through interacting with Methyl-CpG binding domain protein 2 (MBD2), contributing to latency.

Specificity in the HIV gene expression program derives from the combinatorial action of Tat, CRCs, and TFs (Fig. 1A). TFIID and TFIIH along with several other TFs (TFIIA, TFIIB, TFIIE, and TFIIF) bind DNA motifs in the HIV promoter that initiate the pre-initiation complex (PIC) formation (reviewed in [5]). TFIIH phosphorylates Ser7 and Ser5 of the RNA Polymerase II (RNAPII) C-terminal domain (CTD) to activate the polymerase to begin transcription [14]. Recruitment of TFIIH to the PIC has been proposed as the rate-limiting step in the reactivation of latent HIV [15]. Once activated, RNAPII clears the promoter, transcribing the transactivation response element (TAR) RNA, a dynamic stem-loop-bulge secondary structure formed in the first 59 ribonucleotides of HIV transcripts [16], but pauses just after the transcription start site (TSS) due to the occlusion by Nuc-1 and the presence of the negative regulators DRB sensitivity inducing factor (DSIF) and negative elongation factor (NELF) [17]. Stalling of RNAPII at Nuc-1 is relieved by inefficient recruitment of positive transcription elongation factor b (P-TEFb) and host factors, such as NF-κB and BRD4 [18, 19]. Ultimately, some elongation events lead to synthesis of full-length HIV-1 mRNAs, which are spliced to produce Tat. Once Tat accumulates above a certain threshold, it recruits the P-TEFb to the HIV TAR RNA to promote exponential HIV RNA production [20]. P-TEFb is composed of cyclin-dependent kinase 9 (CDK9) and CyclinT1, and CDK9 functions as an early elongation kinase to overcome promoter proximal pausing, through phosphorylation of Ser2 on RNAPII CTD, as well as two elongation factors associated with paused RNAPII (NELF and DSIF) [21]. Through various mechanisms, Tat can thus upregulate HIV transcription by up to 300% more efficiently than cellular activation alone [22, 23].

The initial recruitment of RNAPII to the TSS, PIC formation and the first “burst” of initiation that triggers viral mRNA production are of key importance. These preliminary rounds of transcription initiation allow the production of Tat and the establishment of Tat-TAR feedback loop, since Tat regulates its own transcript production [24, 25]. Tat also promotes transcription in other ways, e.g. by recruiting the super-elongation complex (SEC) [5], histone acetylases [17], and chromatin remodelers that are thought to reposition Nuc-1, the nucleosome located just downstream of the TSS, into a transcriptionally favorable position [4, 26–32].

Given P-TEFb’s central role in regulating eukaryotic gene expression, it is tightly regulated [4, 13]. P-TEFb remains in an inactive state through several mechanisms: (a) predominantly, P-TEFb is found in an inactive state bound to a small nuclear RNA called 7SK snRNA, along with proteins HEXIM, MEPCE and LARP7 [17, 33, 34], and is released from this complex by the host protein BRD4 or HIV Tat protein (Fig. 1A) [13]. (b) P-TEFb activation requires specific cellular signals that are absent in resting cells, such as T cell activation via the T cell receptor (TCR), which can recruit P-TEFb to the HIV promoter. (c) Cyclin T1 is heavily phosphorylated, and this phosphorylated state blocks P-TEFb from interacting with the HIV promoter and initiating transcriptional elongation [33]. Thus, the combination of 7SK snRNA association, absence of activating signals, and Cyclin T1 phosphorylation keeps P-TEFb in an inactive state limiting HIV transcription and contributing to latency.

In this review, we will focus on the role of the human chromatin remodeler protein complexes in modulating HIV-1 transcription. We will discuss recent advances in their functions, novel protein-protein interactions, as well as small molecule inhibitors that may work directly or indirectly, targeting different subunits within the complexes. We also summarize recent advances in the development of small molecule inhibitors targeting the interaction of Tat with its cognate viral TAR RNA.

## Mechanisms regulating nucleosome occupancy and stability

Nucleosomes, the fundamental units of chromatin, directly occlude ~ 80% of the genome, preventing access to transcription factors and enzymes that need access to DNA for transcriptional regulation [35–37]. At cis-regulatory regions, such as the TSSs of active genes promoters, nucleosomes adopt a highly ordered pattern which is orchestrated by numerous mechanisms, including the intrinsic affinity of histones for particular DNA sequences, the concerted activity of multiple ATPase-driven chromatin remodelers [38, 39], and the binding of conventional TFs and RNAPII [35, 40, 41] (Fig. 1). The organization of nucleosomes at the TSS of active genes affects RNAPII recruitment, PIC formation, initiation and pausing, and subsequent transcriptional elongation [40]. A thorough understanding of these mechanisms within the context of latent HIV genomes is crucial for the development of strategies aimed at modulating HIV expression, potentially leading to novel therapeutic approaches for HIV cure.

### HIV genome organization and nucleosome positioning

**Fig. 2:**
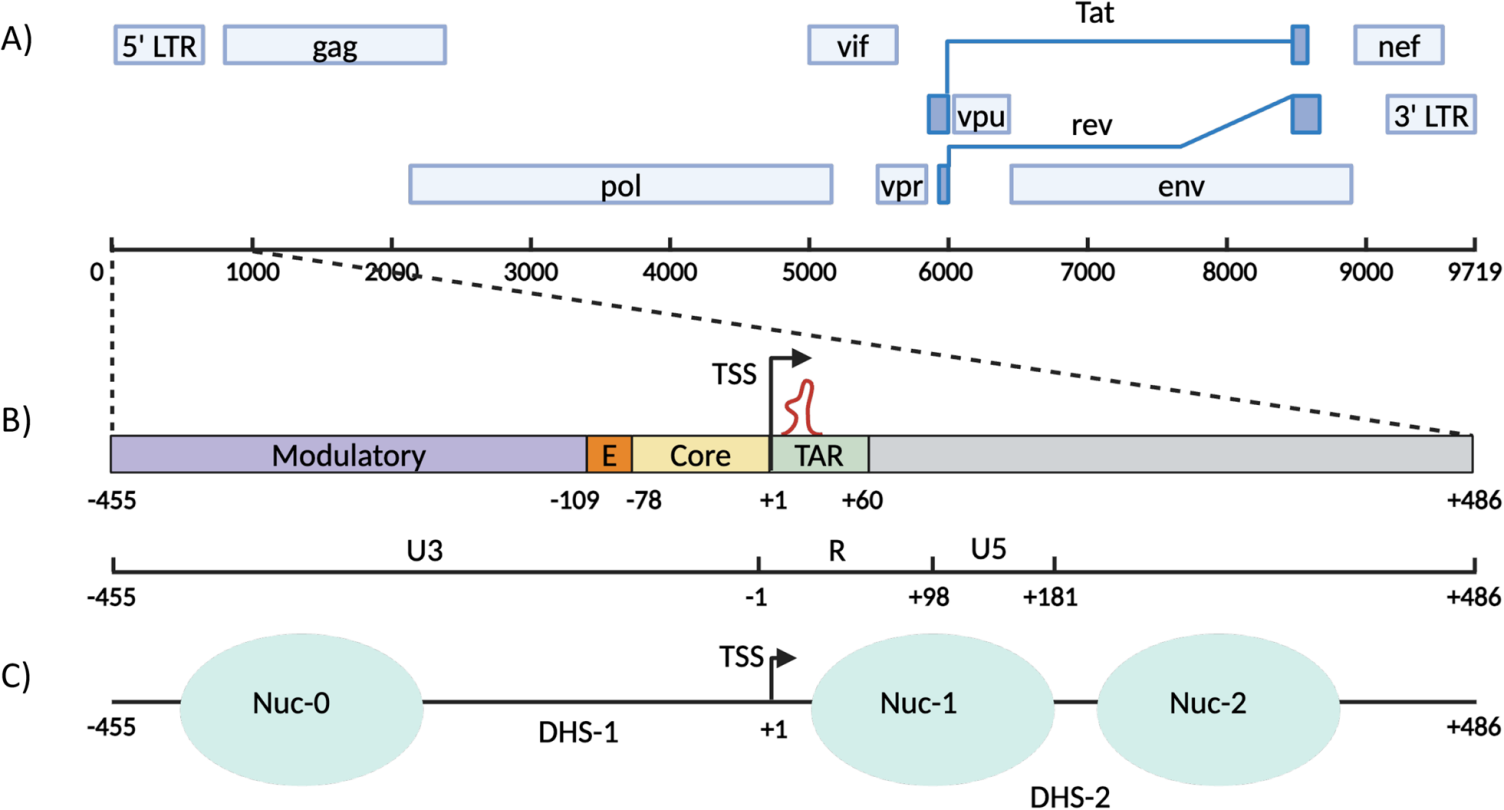
Nucleosomal Structure of the HIV-1 Promoter.** A** The integrated HIV-1 viral genome is 9719 nucleotides long. The genes encoded by the viral genome are depicted relative to their positions within the genome. Each long terminal repeat (LTR) consists of three regions: U3, R, and U5. **B** An expanded view of the 5' LTR highlights four key regions: the modulatory region, the enhancer (E) region, the core promoter, and the transactivation response (TAR) region. **C** The positioning of nucleosomes relative to the HIV transcription start site (TSS) is illustrated. The TSS is indicated by a black arrow, and the nucleosomes (Nuc-0, Nuc-1, and Nuc-2) are represented as mint-colored ovals. The regions between the nucleosomes are labeled as DNase hypersensitivity sites (DHS-1 and DHS-2), signifying areas of increased chromatin accessibility. **B** and **C** Numbers are relative to the TSS.

The HIV-1 genome contains two long terminal repeats (LTRs), located at the 5’ and 3’ ends of the viral genome. Both LTRs can initiate transcription, but viral replication is driven by the activity of the 5’-LTR, which is therefore referred to as the HIV-1 promoter [17]. The structure of the HIV-1 promoter is shown in Fig. 2.

This promoter is divided into U3, R, and U5 regions, which can be further divided into four main segments relative to the TSS: the modulatory region (− 455 to − 104), the enhancer (− 109 to − 79), the core (− 78 to − 1), and the TAR element (+ 1 to + 60) (Fig. 2B) [5, 17, 42].

Nucleosomes are typically positioned as shown in Fig. 2C. Nuc-0 is located upstream of the TSS, while both Nuc-1 and Nuc-2 are located downstream [13, 17]. Nuc-1 is particularly important because it is positioned immediately downstream of the TSS and acts as a barrier for transcriptional elongation. Only upon activation is Nuc-1 remodeled to promote transcription [5, 17]. The DNAse hypersensitive region 1 (DHS-1) is located between Nuc-0 and Nuc-1 and contains the binding sites for most of the cellular transcription factors [17].

To modulate nucleosome occupancy and stability, cells have well-established systems that work collaboratively. Here, we briefly review the post-translational histone modifications and DNA methylation at CpG motifs, while we expand more on specific chromatin remodeling families in the following sections.

### Histone-modifying enzymes

The establishment and maintenance of HIV latency are influenced by modifications to the chromatin structure, which occurs through post-translational changes to histone proteins. Specific enzymes, including HDACs and HMTs, play crucial roles in silencing HIV-1 transcription [13]. While numerous histone post-translational modifications (PTMs), including phosphorylation, glycosylation, sumoylation, ubiquitination, are known to regulate the transcription of cellular genes [43–46], this discussion will primarily focus on histone acetylation and methylation. These modifications are particularly significant in the regulation of HIV.

*a) Histone acetyltransferases (HATs) and histone deacetylases (HDACs)* are the two enzyme families that regulate DNA accessibility by modulating histone acetylation levels [43, 47]. HATs acetylate conserved lysine residues on histone and non-histone proteins by transferring an acetyl group from acetyl-CoA to form ε-N-acetyllysine, while HDACs remove these acetyl groups from both histones and non-histone proteins. Generally, histone H3 and H4 acetylations are associated with transcriptional activation [48], and some examples include H3K9ac and H4K16ac [49, 50]. Originally, it was believed that the negative charge of the acetyl group neutralized the positive charge of histones, thereby loosening the interaction with DNA and increasing the accessibility of DNA to transcription factors [51, 52]. However, recent studies suggest that histone acetylation and other PTMs also generate binding sites for specific protein–protein interactions, such as acetyllysine-binding bromodomain, to facilitate transcription [53].

Research by Zhang et al. has shown that acetylation at K27 of histone H3 (H3K27ac) enhances HIV-1 transcription by directly recruiting the SEC, whereas methylation at R26 of H3 by CARM1 inhibits this recruitment [54]. This group also conducted Chromatin-Immunoprecipitation (ChIP) studies in Jurkat 2D10 cells (an HIV latency cell model) [55] and found that enhanced levels of H3K27ac at Nuc-0 and Nuc-1 correlated with H3K4me3 and RNAPII enrichment, both are indicators of active HIV-1 transcription [54].

The CREB-binding protein (CBP), p300, and the p300/CBP-associated factor (PCAF) are part of the HATs family [29, 56]. In the presence of the HIV Tat protein, these HATs collectively acetylate histones H3 and H4, facilitating chromatin relaxation which enables the binding of other TFs, such as TFIIB and TATA-binding protein (TBP) [57]. Moreover, p300 is known to acetylate the HIV-1 integrase (IN), an activity essential for efficient proviral integration [58].

*b) Histone methyltransferases (HMTs) and histone demethylases (HDMs)* comprise the two main groups of enzymes that regulate histone methylation. This process involves the addition of methyl groups to histones, which can either activate or repress transcription depending on where the methylation occurs on the histones [43]. For instance, activating histone methylations are seen at K4 and K26 of histone H3 (H3K4me and H3K26me) [49, 59], while repressive methylations include occur at K9 and K27 of histone H3 (H3K9me and H3K27me), and at K20 of histone H4 (H4K20me) [50, 60, 61]. Importantly, methylation is generally more stable and longer lasting than acetylation and does not alter the charge of nucleosomes. Specifically, the activating H3K4 methylation has been shown to prevent the repressive methylation at H3K9 [62]. Moreover, H3K4me not only positively cooperates with the acetylation at H3K9 (H3K9ac) but also negatively shows antagonisms with the repressive methylation at the same site (H3K9me) in the context of HIV disease progression [49].

Figure 1B highlights examples of HMTs such as SMYD2 (SET and MYND domain-containing protein 2), which monomethylates H4K20 and recruits L3MBTL1 to facilitate chromatin compaction and promote HIV-1 latency [61]. Additionally, the polycomb repressive complex 2 (PRC2), with its core subunit EZH2, can influence HIV-1 LTR through trimethylating histone H3 at lysine 27 (H3K27me3) [60, 63], making proviruses with this modification less likely to reactivate [63]. Furthermore, SUV39H1, another HMT, contributes to heterochromatin formation by trimethylating H3K9 [63, 64].

In conclusion, histone-modifying enzymes such as HATs, HDACs, HMTs, and HDMs play vital roles in controlling HIV-1 transcription by affecting chromatin structure. These enzymes are responsible for post-translational modifications that influence gene expression and accessibility—HATs enhance transcription by relaxing chromatin, while HDACs inhibit it by removing acetyl groups. Methylation events by HMTs can both activate or suppress gene expression, based on the context. These dynamic modifications are critical for managing viral latency and reactivation, underscoring their potential as targets in HIV-1 therapeutic strategies. Continuously expanding our molecular understanding is essential for effective HIV-1 management and potential eradication.

### DNA methylation at CpG motifs

Similar to histone methylation, DNA methylation can also influence the availability of DNA for transcription. Under transcriptionally active conditions, CpG islands (CGIs) are unmethylated, and histones H3/H4 are acetylated, while H3 is methylated [65]. When DNA is methylated on CGIs, promoters containing CGIs can be stably silenced long-term either through direct inhibition of transcription factors binding to methylated DNA, or through methyl-binding domain (MBD) protein-mediated repressive events, such as MeCP2, which in turn recruit other modifiers including HDACs and HMTs, to reinforce the repressive chromatin state [65, 66]. In relation to HIV-1 latency, Watanabe's group has shown that CpG sites in the 5'-LTR of latent HIV provirus are hypermethylated [67]. By measuring the CpG methylation levels in different cell lines, they also found that the level of methylation is inversely correlated with the viral gene expression at basal levels, indicating that the more silenced the provirus is, the higher the level of DNA methylation at CpG motifs [67].

Later in 2009, the Hirsch group further elucidated this correlation in Jurkat clones, revealing that while high levels of methylation at the 5’LTR are not essential for HIV promoter silencing, they may nonetheless serve as a mechanism to maintain viral latency [68]. However, subsequent studies by Blazkova et al. found that in resting CD4 + T cells isolated from blood of PLWH on ART, the 5’LTR was rarely observed to be highly methylated [69]. Consequently, the specific roles of DNA methylation at HIV promoter CpG motifs remain unclear, underscoring the need for further investigation into this topic.

## Chromatin remodeler protein families

Chromatin remodelers are large, multiprotein complexes that use the energy of ATP hydrolysis to mobilize, eject, or restructure nucleosomes. Eukaryotic/human cells contain four families of chromatin remodeling complexes based on the similarities and differences of the ATPase subunits: the SWI/SNF, ISWI, CHD, and INO80 families. All four families consist of a core ATPase and additional modulatory subunits, and they are collectively known as ATP-dependent chromatin remodelers. By restructuring and repositioning histones, chromatin remodelers alter chromatin accessibility, thereby influencing DNA transcription and repair.

**Fig. 3:**
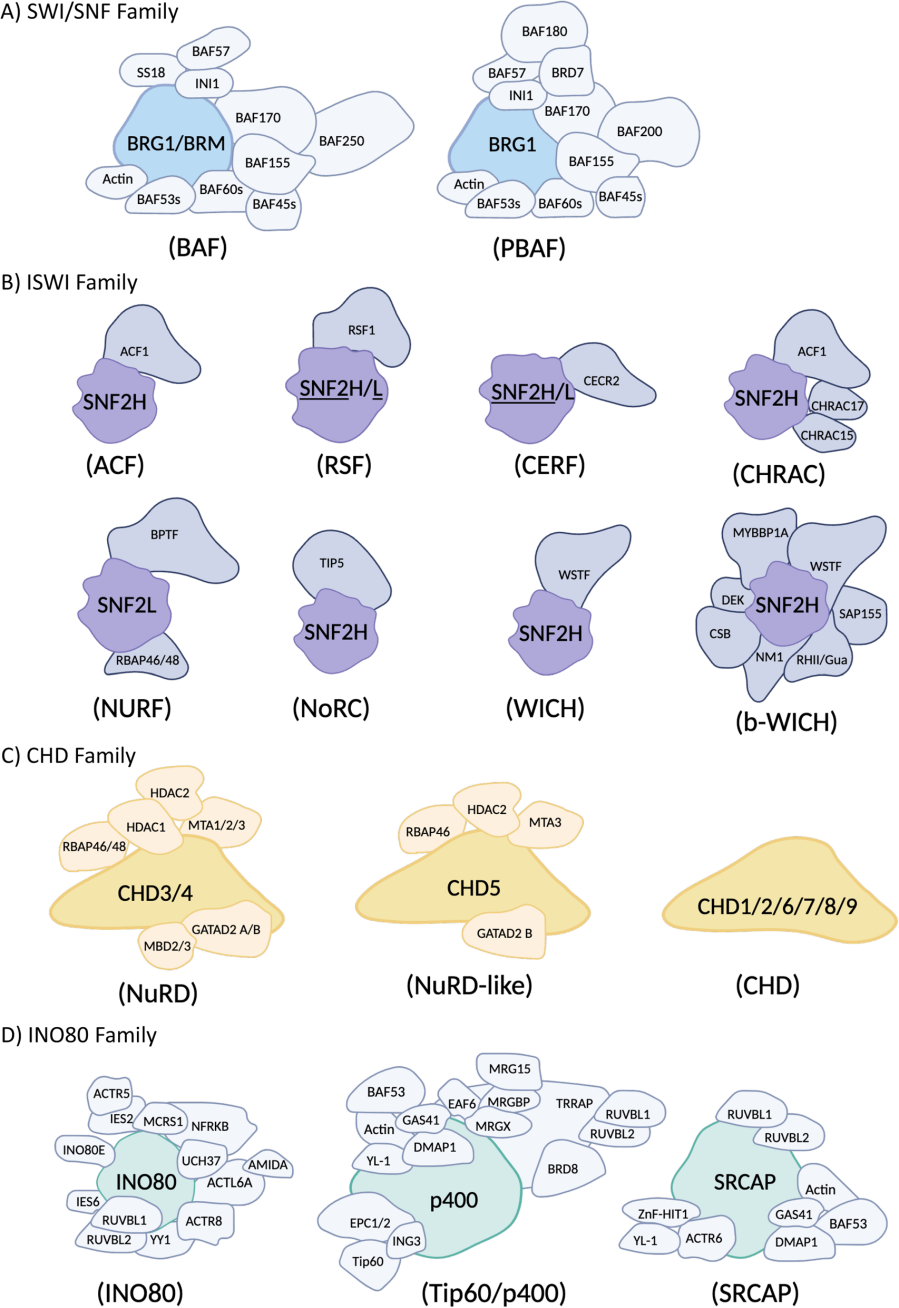
Chromatin Remodeler Families and Their Associated Core ATPase Subunits. This figure presents the four major chromatin remodeler families, highlighting their members and associated core ATPase subunits. The central, brightly colored subunit in each illustration represents the core ATPase, while the size of each shape depicts the relative size of each subunit within the complex. **A** BAF and PBAF complexes and their relative subunits. **B** ISWI family members and their subunits. The preferred ATPase subunits of the RSF and CERF complexes are underlined. **C** CHD family members and their subunits. **D** INO80 family members and their subunits.

*1. The SWI/SNF (SWItching defective/Sucrose NonFermenting) family* includes two independent complexes: BAF and PBAF (Fig. 3A). Although these two complexes share up to 9 subunits, including the ATPase (Table 1), their functions are relatively different. BAF has been reported to have repressive activity by directly associating with the HIV LTR and remodeling Nuc-1 into a less favored position for RNAPII elongation, whereas PBAF is recruited during HIV transcriptional activation and is necessary for Tat-dependent transcription of HIV (Fig. 1) [27, 70].

**Table 1:** Complexes in the SWI/SNF Family and Their Subunits. Starred subunits are the common ones shared by the BAF and PBAF complexes.

Complex	SWI/SNF	
	BAF	PBAF
ATPase	BRG1/BRM	BRG1
Other subunits	ARID1A/B (BAF250)	ARID2 (BAF200)
	INI1*	INI1
	BAF45 A/B/C/D*	BAF45 A/B/C/D
	BAF53 A/B*	BAF53 A/B
	BAF57*	BAF57
	BAF60 A/B/C*	BAF60 A/B/C
	BAF155*	BAF155
	BAF170*	BAF170
	Actin*	Actin
	SS18	BAF180
		BRD7

BRG1 (Brahma-related gene-1) also known as SMARCA4, is the core ATPase subunit shared by BAF and PBAF complexes (Fig. 3A**, **Table 1). Within the BAF complex, BRM (or SMARCA2) can substitute for BRG1, and these two proteins are mutually exclusive (Fig. 3A**, **Table 1). In several cancer types, BRG1 deficiency is associated with chemotherapy resistance, which worsens if BRM is also epigenetically silenced through mutations [71]. BRG1/BRM drives the production of IP3R3 (inositol 1,4,5- trisphosphate receptor type 3) protein, which forms Ca^2+^ channels on the endoplasmic reticulum (ER) and is important for intracellular homeostasis and cellular apoptosis. BRG1/BRM deficiency can impair the function of these calcium channels and programmed cell death [71]. Conrad et al. discovered that the short isoform of bromodomain and extra-terminal (BET) family member BRD4 (BRD4S) binds directly to BRG1 within the BAF complex and promotes HIV-1 latency [72].

INI1, also known as hSNF5, BAF47, or SMARCB1, is a component common to both the BAF and PBAF complexes (Table 1). Research by Easley et al. and Mahmoudi et al. revealed that the depletion of INI1 and BRG1 prevents Tat-mediated activation of HIV [32, 73]. Subsequently, INI1 was discovered to interact with various proteins, including HIV-1 IN and c-myc. The Rpt1 domain of INI1 (aa 186–245) is essential for binding to HIV-1 IN and inhibiting HIV-1 replication [74, 75]. Moreover, IN was shown to associate with the TAR element through an AlphaScreen assay [76]. Dixit et al., using computational modeling, proposed that INI1 inhibits HIV-1 IN by competitive inhibition, specifically through mimicking the TAR RNA structure [75]. This finding may appear contradictory to earlier findings by Mahmoudi et al.; however, it is possible that Mahmoudi's data is more pertinent to the PBAF complex, whereas the latter findings relate more closely to the BAF complex.

As one of the most studied chromatin regulatory families in the context of HIV, SWI/SNF has demonstrated its importance in modulating HIV-1 latency. The BAF complex maintains latency via BRD4, and the PBAF complex facilitates Tat-dependent HIV activation.

*2. The ISWI (Imitation SWItch) family* includes multiple members, some of which operate with as few as 2 subunits, while others require up to 8 subunits including the ATPase (as shown in Fig. 3B and Table 2). In the cases of RSF and CERF complexes, either SNF2H or SNF2L may serve as the core ATPase; however, previous studies suggest that RSF may prefer SNF2H, whereas CERF may favor SNF2L [43]. The ISWI family plays a crucial role in regulating nucleosomal spacing. Within this family, some members, such as ACF and CHRAC, promote chromatin compaction, while others like NURF, inhibit chromatin assembly [43]. As for now, not much is known regarding the ISWI family, which is indicating the need for further investigations and discovery.

**Table 2:** Complexes in the ISWI Family and Their Subunits. For RSF and CERF, the preferred ATPase is shown in **bold** letters.

Complex	ISWI							
	ACF	RSF	CERF	CHRAC	NURF	NoRC	WICH	b-WICH
ATPase	SNF2H	**SNF2H**/L	**SNF2**H/**L**	SNF2H	SNF2L	SNF2H	SNF2H	SNF2H
Other subunits	ACF1	RSF1	CECR2	ACF1	BPTF	TIP5	WSTF	WSTF
				CHRAC15	RBAP46/48			DEK
				CHRAC17				CSB
								NM1
								SAP155
								MYBBP1A
								RHII/Gua

*3. The CHD (Chromodomain, Helicase, DNA binding) family* is divided in three groups: NuRD, NuRD-like, and CHD (Fig. 3C). NuRD-like is recognized as a distinct group due to its different core ATPase compared to NuRD; however, it shares all other subunits with NuRD, as specified in Table 3. The NuRD groups can suppress gene expression using HDAC subunits. In contrast, the CHD group, containing only the ATPase member, facilitates gene expression by repositioning nucleosomes on the chromatin.

**Table 3:** Complexes in the CHD Family and Their Subunits.

Complex	CHD		
	NuRD	NuRD-like	CHD
ATPase	CHD3/4	CHD5	CHD1/2/6/7/8/9
Other subunits	HDAC1	HDAC2	
	HDAC2	GATAD2 B	
	MTA1/2/3	MTA3	
	RBAP46/48	RBAP46	
	GATAD2 A/B		
	MBD2/3		

MTA proteins in the NuRD complexes play diverse roles across different cell lines. In T cells, for example, MTA1 directly interacts with BCL11B (also called CTIP2), a transcription factor that represses the HIV promoter, with the first 45 amino acids of BCL11B being essential for this interaction [77]. BCL11B is also present in microglial cells, where it can circumvent the NuRD complex by directly recruiting HDACs [78]. In addition to MTA1, the NuRD complex contains MTA2 and MTA3. For instance, in HeLa cells, the transcription factor YY1 and the immunophilin FKBP25 exclusively bind MTA2 [79], while MTA3 interacts with BCL6, a transcriptional repressor, in germinal center B cells [80].

In 2011, Gallastegui et al. discovered that in intron-integrated HIV latency models (J-Lat E27 and A2), silencing CHD1 promoted the reactivation of latent HIV [81]. This effect was further enhanced by stimulation with latency reversing agents (LRAs) such as TSA and TNF-α [81]. This finding suggested that CHD1 might act as a repressor of HIV expression. However, a few years later, Rodgers et al. reported that CHD1 and CHD2 could instead function as enhancers of HIV-1 expression [82]. Their study using RNAi depletion also revealed that CHD2 might compensate for the loss of CHD1 expression [82]. This conflicting data suggests that the role of CHD proteins in HIV latency and reactivation is complex and may be context-dependent. Further research is needed to clarify the specific mechanisms by which CHD1 and CHD2 regulate HIV expression.

Meanwhile, CHD9, another member of the same family, exerts a repressive effect on HIV-1 [83]. Roling et al. demonstrated that CHD9 could directly bind to the HIV-1 promoter and repress HIV-1 transcription [83], an effect that is reversible with PMA (phorbol 12-myristate 13-acetate) treatment, a compound known to stimulate HIV expression [83].

The research thus far suggests that the CHD group plays a more significant role in modulating HIV transcription compared to the NuRD complexes. However, further investigation is essential to fully elucidate the specific roles of CHD family members in the context of HIV biology.

*4. The INO80 (INOsitol requiring 80) family* consists of three different complexes: INO80, SRCAP, and TIP60/p400 (Fig. 3D). Among these complexes, RUVBL1 and 2 are the only subunits shared by all three. Additionally, there are five more subunits that are shared between TIP60/p400 and SRCAP complexes (Table 4) [84, 85]. All three complexes in the INO80 family play critical roles in DNA damage response and repair processes [43]. The INO80 complex is primarily involved in promoting transcription, facilitating DNA repair, and restructuring nucleosomes to maintain genome integrity [43]. The Tip60/p400 complex is crucial for marking sites of DNA damage to ensure they are appropriately targeted for repair [43]. Meanwhile, the SRCAP complex responds to DNA damage by regulating the replacement of histones, thereby aiding in the restoration of chromatin structure and function [43].

**Table 4:** Complexes in the INO80 Family and Their Subunits.

Complex	INO80		
	INO80	Tip60/p400	SRCAP
ATPase	INO80	p400	SRCAP
Other subunits	RUVBL1	RUVBL1	RUVBL1
	RUVBL2	RUVBL2	RUVBL2
	MCRS1	TRRAP	GAS41
	AMIDA	Tip60	BAF53
	ACTL6A	BRD8	DMAP1
	YY1	BAF53	YL-1
	IES6	EPC1/2	ARP6 (ACTR6)
	IES2	YL-1	ZnF-HIT1
	UCH37	GAS41	Actin
	NFRKB	DMAP1	
	INO80E	ING3	
	ACTR5	Actin	
	ACTR8	MRG15	
		EAF6	
		MRGX	
		MRGBP	

Tip60 protein, also called KAT5 (lysine acetyltransferase 5), is a member of nuclear HATs, and it was initially discovered as a coactivator of HIV-1 Tat activity in the HeLa cell line [86, 87]. However, the function of Tip60 in transcription was later found to be gene-dependent [88, 89]; while Tip60 can be a coactivator of Tat in HeLa cells, its activity can also be inhibited by Tat in Jurkat cells [89]. Tat inhibits Tip60 through interfering with the HAT activity [87] and inducing selective degradation of Tip60 [90]. This neutralization of Tip60 is key for the protection of HIV-infected cells from DNA damage-induced apoptosis [90].

In essence, although each complex within the INO80 family has specialized functions, they collectively contribute to maintaining genomic stability by participating in various aspects of the DNA damage response and repair mechanisms. Further insights into the specific roles and interactions of these complexes may provide a deeper understanding of their contributions to cellular homeostasis and disease processes.

## Small molecules targeting chromatin remodelers and related proteins—SWI/SNF family

JQ1 is a potent pan-BET inhibitor discovered in 2010 by Filippakopoulos et al. [91]. In 2012, its potential as an HIV therapeutic agent was explored, as BRD4 is known to promote HIV latency by competing with Tat for P-TEFb recruitment to RNAPII [92, 93]. The results showed that JQ1 activated HIV by competitively binding to BRD4 and promoted BRD4 dissociation from Tat. This allowed for increased Tat binding to the promoter, thereby activating HIV transcription [92].

**Fig. 4:**
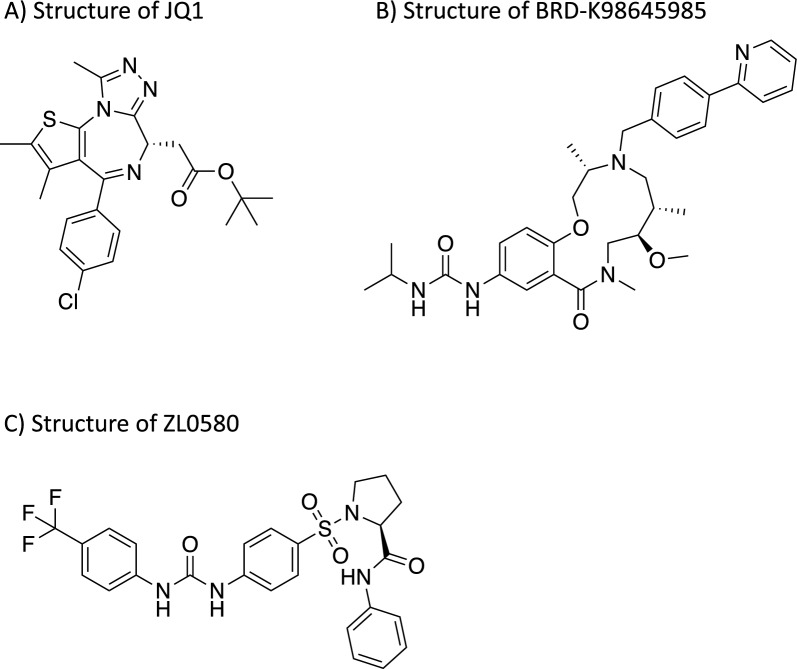
Examples of Compounds Targeting the SWI/SNF Family of Chromatin Remodelers. **A** Chemical structure of JQ1. **B** Chemical structure of BRD-K98645985. **C** Chemical structure of ZL0580.

Recent findings by Ott’s group indicate that the BRD4S directly interacts with BRG1 [72]. Building on this finding, Conrad and colleagues tested JQ1 (Fig. 4A) and discovered that JQ1 can mediate HIV-1 latency reversal independently of Tat by targeting the repressive BAF complex, with BRD4S showing a high responsiveness to JQ1 treatment [72, 91]. However, it is important to note that JQ1 has many other targets within the BET family [91]. Consequently, researchers have sought to develop more potent and specific molecules for modulating the BAF complex.

BAF250 is encoded by the ARID1 gene and has two paralogs, BAF250a (ARID1A) and BAF250b (ARID1B) [94]. Although these paralogs share similar structures, including ARID and ARM domains, they serve different functions: BAF250b is reported as a tumor suppressor protein, whereas BAF250a is essential for repressing latent HIV [27, 94]. In 2018, Marian et al. discovered BRD-K98645985 (Fig. 4B) through high-throughput screening (HTS). Via Structure–Activity Relationship (SAR) studies, they identified several potent analogs targeting ARID1A [95]. These compounds function as LRAs, binding to the BAF250a subunit and increasing chromatin accessibility by preventing Nuc-1 from shifting to the unfavorable position, thereby reversing HIV-1 latency [95].

In addition to inhibiting the BAF complex to reverse latency, researchers have also explored strategies to further enhance latency as a potential treatment approach. In 2019, ZL0580 was identified through structure-based drug design (Fig. 4C). This compound was found to suppress HIV-1 both in vitro and ex vivo in CD4^+^ T cells and peripheral blood mononuclear cells (PBMCs) of either viremic or aviremic PLWH [96]. When combined with ART, ZL0580 also led to a delay in viral rebound in PBMCs of PLWH [96]. When tested in J-Lat cells, ZL0580 was found to reduce Tat recruitment to the HIV promoter while increasing the recruitment of BRD4 to the promoter and inducing chromatin remodeling for a more repressive environment in the HIV LTR [96]. Unlike JQ1, which non-selectively binds to both bromodomain 1 and 2 (BD1 and BD2) of BRD4 and other BET family members, ZL0580 is over six times more selective for BD1 than BD2 and about six to eleven times more selective for BRD4 over other BET proteins [96]. This selectivity minimizes non-specific binding, making ZL0580 a promising BRD4 BD1 modulator that promotes HIV-1 latency.

To summarize, research on small molecule modulators has primarily focused on the SWI/SNF family of chromatin remodelers, particularly members of the BAF complex. The core ATPase, BRG1, is a prime target due to its direct association with BRD4 proteins. While both JQ1 and ZL0580 target BRD4, JQ1 can reverse HIV latency through dissociating BRD4 from competing with Tat, whereas ZL0580 can further promote HIV-1 latency through increasing BRD4 recruitment to limit Tat from binding to the HIV promoter and inducing remodeling for a repressive LTR. BAF250 is another key target for latency reversal. Considering that the PBAF complex, the Tip60/p400 complex, and the CHD family also play significant roles in modulating HIV-1 transcription, there is substantial potential for the discovery of new small molecules and novel therapeutic approaches against HIV as we further our understanding of these chromatin remodelers.

## Exploiting small molecules to inhibit HIV transcription through targeting Tat-TAR interaction

**Fig. 5:**
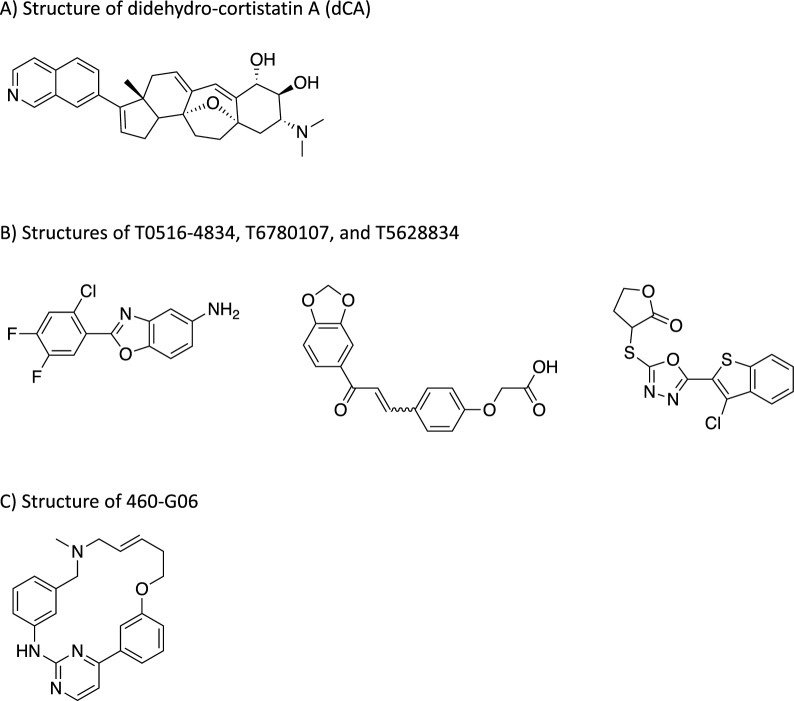
Examples of Compounds Inhibiting the Tat-TAR Interaction. **A** Chemical structure of didehydro-cortistatin A (dCA). **B** Chemical structure of T0516-4834, T6780107, and T5628834 (from left to right). **C** Chemical structure of 460-G06.

The reliance of HIV on the Tat protein for its transcriptional activity has spurred the development of highly potent inhibitors against Tat. In 2012, Mousseau et al. discovered didehydro-cortistatin A (dCA), an analog of the naturally occurring compound Cortistatin A, isolated from a marine sponge, which acts as a powerful Tat inhibitor with sub-nanomolar IC_50_ (Fig. 5A) [97]. To gain a deeper understanding of dCA's mechanism of action on Tat, Mediouni et al. in 2019 performed a series of biophysical assays and uncovered that dCA binds to Tat's basic domain, thereby blocking its interaction with TAR RNA [98]. To test dCA’s effects when combined with ART treatment, Li et al. tested dCA in the chronically infected HeLa-CD4 cell model under ART treatment, while SAHA was used as the LRA [70]. The results showed that dCA promoted a greatly repressive chromatin environment with reduced histone acetylation and PBAF recruitment, and increased Nuc-1 occupancy, which rendered the stimulation by SAHA less effective at reactivating the virus [70].

Kessing et al. later evaluated the efficacy of dCA in conjunction with ART ex vivo in CD4 + T cells isolated from PBMCs of PLWH [99]. Compared to ART treatment alone, the group receiving both dCA and ART experienced accelerated HIV-1 suppression, with no viral rebound for at least seven days post-treatment interruption, and minimal to no viral rebound even after subsequent stimulation [99].

Further investigations by the Valente group tested the effects of dCA on viral persistence within lymphoid tissues in bone marrow-liver-thymus (BLT) humanized mice. Under ART treatment, the dCA-treated mice exhibited significantly reduced cell associated viral RNA copies across all lymphoid tissues tested, along with a delayed viral rebound following treatment interruption [99]. Moreover, given that Tat is known to cross the blood–brain barrier (BBB) and induce neurotoxicity [100], dCA's BBB permeability was investigated in a pharmacological study in mice along with Tat’s potentiation of cocaine-mediated psychological stimulation. The results confirmed that dCA can traverse the BBB and significantly reduce Tat-induced cocaine potentiation, warranting further research into its potential effects on Tat-related neurotoxicity [101].

Several research groups have discovered small molecules with diverse structures capable of inhibiting Tat-induced transcription by directly targeting the TAR RNA. In 2021, Nekhai's group screened over two million compounds in the Enamine library, identifying T0516-4834, T6780107, and T5628834 as effective HIV-1 inhibitors (Fig. 5B) [102]. While both T5628834 and T0516-4834 showed reduced levels of CDK9 and Cyclin T1 association, T0516-4834 emerged as the most promising candidate, with selectivity for Tat-induced transcription and an IC_50_ of 0.3 μM in a single-round HIV-1 inhibition assay [102].

Another noteworthy TAR-binding small molecule inhibitor, 460-G06, was discovered through a time-resolved fluorescence resonance energy transfer (TR-FRET) assay screening over 39,000 compounds (Fig. 5C). With an IC_50_ of 0.011 μM for Tat activity and an IC_50_ of 0.013 μM for HIV-1 infectivity, 460-G06 inhibits the Tat-TAR interaction by directly binding to TAR RNA and efficiently dissociating Tat from TAR [103].

Overall, research into small molecules targeting the Tat-TAR interaction has grown substantially in recent decades. While a variety of biopolymers and small molecules targeting the Tat-TAR interaction and the Tat-P-TEFb have been identified (reviewed in [104]), dCA remains the most promising Tat-specific inhibitor demonstrated by its sub-nanomolar IC_50_ and efficacy in both humanized mice and in vitro studies using PBMCs from people living with HIV.

## Conclusion

While significant advancements have been made in understanding HIV-1 transcription mediated by chromatin remodelers, detailed knowledge remains predominantly focused on the SWI/SNF family. Research on the CHD family is comparatively limited, and even less is known about the ISWI and INO80 families.

From what is understood about gene transcription stages in general, the ISWI family is reported to play a role in transcriptional initiation [105]. Similarly, the CHD family modulates initiation by inducing changes in nucleosome structure and density [43, 106]. During the subsequent elongation stage, the INO80 family aids in repositioning Nuc-1 to promote transcriptional elongation [107]. The SWI/SNF family also plays a critical role in this stage, with the PBAF complex enhancing transcriptional elongation and the BAF complex often inhibiting it through similar nucleosome remodeling mechanisms [5, 43].

Here we reviewed the recent advances concerning the SWI/SNF and CHD families in the context of HIV-1 transcription. We highlighted small molecule modulators of the BAF complex that hold potential for further modification and development into clinical trial-compatible compounds. Additionally, we discussed recently discovered small molecule modulators of the Tat-TAR interaction. With continued research and innovation, there is hope to achieve the complete silencing or eradication of HIV-1.

## Data Availability

No datasets were generated or analysed during the current study.

## Abbreviations

PLWH: People living with HIV
ART: Antiretroviral therapy
TFs: Transcription factors
CRCs: Chromatin regulatory complexes
PIC: Pre-initiation complex
RNAPII: RNA polymerase II
CTD: C-terminal domain
TAR: Transactivation response element
TSS: Transcription start site
DSIF: DRB sensitivity inducing factor
NELF: Negative elongation factor
P-TEFb: Positive transcription elongation factor b
CDK9: Cyclin-dependent kinase 9
SEC: Super-elongation complex
TCR: T cell receptor
LTRs: Long terminal repeats
DHS-1: DNAse hypersensitive region 1
PTMs: Post-translational modifications
HATs: Histone acetyltransferases
HDACs: Histone deacetylases
H3K27ac: Acetylation at K27 of histone H3
ChIP: Chromatin-Immunoprecipitation
HMTs: Histone methyltransferases
HDMs: Histone demethylases
H3K4me: Methylation at K4 of histone H3
H3K26me: Methylation at K26 of histone H3
H3K9me: Methylation at K9 of histone H3
H3K27me: Methylation at K27 of histone H3
H4K20me: Methylation at K20 of histone H4
SMYD2: SET and MYND domain-containing protein 2
PRC2: Polycomb repressive complex 2
CGIs: CpG islands
MBD: Methyl-binding domain
SWI/SNF: Switching defective/sucrose nonfermenting family
BRG1: Brahma-related gene-1
IP3R3: Inositol 1,4,5- trisphosphate receptor type 3
ER: Endoplasmic reticulum
BET: Bromodomain and extra-terminal family
BRD4S: Short isoform of BRD4
ISWI: Imitation switch family
CHD: Chromodomain, helicase, DNA binding family
LRAs: Latency reversal agents
PMA: Phorbol 12-myristate 13-acetate
INO80: Inositol requiring 80 family
HTS: High-throughput screening
SAR: Structure-activity relationship
PBMCs: Peripheral blood mononuclear cells
BD1: Bromodomain 1
BD2: Bromodomain 2
dCA: Didehydro-cortistatin A
BLT: Bone marrow-liver-thymus
BBB: Blood-brain barrier
TR-FRET: Time-resolved fluorescence resonance energy transfer
GTFs: General transcription factors
TBP: TATA-binding protein
7SK snRNA: 7SK small nuclear RNA
DNMTs: DNA methyltransferases
MBD2: Methyl-CpG binding domain protein 2
